# Effect of Particulate Matter Concentration on Changes in Physiological Fatigue Variables with Resistance Exercise

**Published:** 2019-12

**Authors:** Sang Soo KIM, Woo Yeul BAEK, Sung Bum JU

**Affiliations:** 1. Department of Physical Education, Keimyung University, Daugu, South Korea; 2. Department of Sport Management, Kyonggi University, Suwon, South Korea; 3. Department of Physical Education, Busan National University of Education, Busan, South Korea

## Dear Editor-in-Chief

High particulate matter (PM) concentration during exercise application can adversely affect cardiopulmonary and immune functions and potentially cause health problems (1). Indoor sporting activities, such as football, basketball, badminton, and fitness training, led to about three times faster breathing than being at rest; because PM_10_ concentration indoors was about twice as high as that outdoors, exercise at a gym was accompanied by PM_10_ that was effectively about six times as high as that outdoors (2). Nevertheless, recently no significant change was found in exercise performance, in pulmonary functions, or in the biological markers of inflammation with mild and acute exposure to PM_2.5_ when acute bouts of vigorous exercise were carried out at low ambient PM_2.5_ concentrations (<12 μg/m^3^) and at an air quality index (AQI) ranking of “yellow” (3). Exposure to PM_10_ and O_3_ was found to increase general and physical fatigue according to the results of an MFI-20 questionnaire, which can rate subjective fatigue (4). Lactate dehydrogenase (LDH) and creatine phosphokinase (CPK) were chosen to be used for evaluation of the physiological changes caused by exercise load on the body. Both LDH and CPK are used as indicators of fatigue types on the basis of the process of energy metabolism (5).

We aimed to determine the effects of PM concentration on LDH and CPK with resistance exercise and provide information about the degree of physiological fatigue variables.

Eight men, who were college students in their twenties (Mean ± SD: 25.0 ± .6 y; 1.74 ± 0.05 m; 69.3 ± 6.2 kg), participated in the experiment and were asked to do resistance exercise on days at low (PM_2.5_ : <15 μg/m^3^, PM_10_ : <30 μg/m^3^) and high PM concentration (PM_2.5_ : 35–75 μg/m^3^, PM_10_ : 80–150 μg/m^3^) as determined by a portable PM sensor (KoRes, KOREA) as well as by Air Korea, a website that provides a comprehensive air-quality index. Resistance exercise lasted 80 minutes: 60 minutes for the main exercise. Exercise intensity was 60% to 70% of 1RM; three sets for each of ten events were provided, with each set having 7–9 sessions; and inter-session recess lasted 1 minute. Blood sampling was performed at pretest before resistance exercise, immediately after the end of resistance exercise, and at 15 min of the recovery phase. The blood fatigue variables, serum LDH and serum CPK, were analyzed by LDLP and IFCC.

For statistical processing, the mean and standard deviation were estimated using an SPSS 21 program (Chicago, IL, USA); simple and repeated methods were used for differences by time in each group. The statistical significance level was set at 5%.

LDH was significantly higher at 10 minutes of the recovery phase than before exercise at high PM concentration (*P*<.05), but there was no significant difference by time at low PM concentration. Moreover, no significant difference was found in CPK by time between high and low PM concentrations. This is probably the first research on the changes in the physiological fatigue variables LDH and CPK for prediction of exercise fatigue with resistance exercise based on PM concentration (Table 1).

**Table 1:** Particle matter concentration and changes in LDH and CPK with resistance exercise

***Variables***	***Phase Group***	***Before exercise***	***Immediately after exercise***	***10 min in recovery***	***Post-hoc***
LDH(U/L)	High PM	223.50±63.17	280.66±125.36	273.83±85.00	Before<10 min in recovery
	Low PM	217.66±41.00	274.78±78.69	253.66±70.29	-
CPK(U/L)	High PM	157.50±71.37	190.33±90.89	191.66±100.24	Before<immediately after exercise Before<10 min in recovery
	Low PM	175.00±53.49	200.33±65.54	195.16±63.71	Before<immediately after exercise Before<10 min in recovery

Data are presented as mean ± standard deviation

On those days at high PM concentration, physical activity could increase PM inhalation with increase in the respiration rate and respiratory quotient per minute, compared with rest (6). Resistance exercise is characterized by higher, stable blood pressure than at rest. A correct pattern of breathing is used to minimize the rise in blood pressure: exhalation for lifting weight and in halation for lowering it (7). This type of breathing for resistance exercise is expected to have exerted clear effects on the exercise fatigue variables at high PM concentration.

In conclusion, resistance exercise led to delayed recovery of LDH at high PM concentration; PM inhalation can increase the physiological burden of resistance exercise because of the specific type of breathing and heavier burden of breathing based on exercise load. Therefore, attention should be paid to the possibility that recovery of exercise fatigue variables can be delayed.
